# Comment letter: reflections on “Perioperative metabolomic alterations predict postoperative complications and hospital stay in colorectal cancer patients”

**DOI:** 10.1097/JS9.0000000000005047

**Published:** 2026-03-12

**Authors:** Yang Li, Jie-Min Huang, Rong Shi, Zu-Qing Chen

**Affiliations:** a Department of Anorectal surgery, The Affiliated People’s Hospital of Fujian University of Traditional Chinese Medicine, Fuzhou, China; b Chen Minfan’s TCM Master Inheritance Studio, Fuzhou, China

*Dear Editor,*

We greatly appreciate Liu *et al*’s study on perioperative metabolomics and surgical outcomes in colorectal cancer (CRC) patients, recently published in the International Journal of Surgery^[^1^]^. This work effectively integrates clinical data with high-throughput nuclear magnetic resonance (NMR)-based metabolomic profiling, identifying key metabolic predictors of postoperative complications and hospital stay in 243 CRC patients, and bridges basic research and clinical practice. As researchers in surgical metabolomics and outcome prediction, we admire the rigorous design and impactful findings, and offer suggestions to enhance reproducibility, clinical relevance, and translational potential.

First, standardizing metabolomic methodologies is critical for cross-study validation. While NMR ensures quantitative precision, metabolomic results are sensitive to technical variables (sample collection, extraction, detection, and modeling) that may cause heterogeneity. Gas chromatography-mass spectrometry (GC-MS) and ultra-high-performance liquid chromatography (UPLC)-based techniques – well-validated in CRC metabolomics^[^2^]^ – complement NMR in metabolite coverage and sensitivity for specific compound classes (e.g., amino acids, fatty acids). A brief comparison of NMR results with UPLC-validated markers (e.g., guanosine, sphingosine) or pathway overlaps (e.g., arginine biosynthesis, pyrimidine metabolism) identified in prior CRC studies^[^2^]^ would contextualize findings. Aligning sample collection timelines with international guidelines and disclosing detailed protocols (as in studies analyzing perioperative metabolomic trajectories^[^3^]^) will minimize bias and boost biomarker reproducibility.

Second, refined clinical stratification can optimize predictive precision. Perioperative metabolic responses vary by surgical approach (laparoscopic vs open) and tumor characteristics (stage, location, and differentiation), which may yield subtype-specific metabolic signatures – consistent with findings that minimally invasive surgery correlates with distinct metabolic and inflammatory profiles^[^4^]^. Additionally, models integrating metabolomic markers with clinical factors (e.g., cortisol, C-reactive protein) have shown strong performance in predicting surgical outcomes^[^1^]^, similar to the integrated frameworks previously proposed. Incorporating such clinical-metabolic integration, alongside perioperative management details [e.g., Enhanced Recovery After Surgery (ERAS) adherence], will enhance adaptability to individual differences in clinical practice.

Third, applying the GRADE framework and extending follow-up strengthens evidence quality. The GRADE framework^[^5^]^ systematically evaluates bias risk, consistency, and precision – key for clinical translation, as demonstrated in metabolomic studies of CRC prognosis^[^2^]^. While short-term outcomes (complications, hospital stay) are relevant, long-term outcomes (delayed anastomotic leakage, sustained gastrointestinal function) matter equally: recent work shows postoperative metabolic alterations persist for up to 1 year and correlate with recovery trajectories^[^3^]^. Extending follow-up to 6–12 months will validate biomarker durability and uncover long-term recovery-related targets.

Finally, exploring mechanistic pathways bridges prediction and intervention. Markers like 2-aminobutyric acid (linked to inflammation) and Very-Long-Chain Polyunsaturated Lipid (V3PL) ratio (reflecting lipid transport) lack clear biological mechanisms, but prior CRC metabolomic studies offer clues: for example, postoperative restoration of specific metabolites to healthy levels correlates with improved tissue repair^[^2^]^, and metabolic pathway modulation enhances recovery^[^4^]^. Elucidating such pathways – similar to how metabolome traits were linked to postoperative CRC recovery^[^3^]^ – will transform predictors into therapeutic targets, enabling interventions like nutritional modulation to improve outcomes.

In conclusion, Liu *et al*’s study^[^1^]^ advances perioperative metabolomic research for CRC. Our suggestions aim to complement their valuable work. We believe refined biomarkers could play a pivotal role in personalized perioperative care, and eagerly anticipate the authors’ responses and future breakthroughs.

## Ethical approval

Not applicable.

## Consent

Not applicable.

## Data Availability

This letter is a commentary on published academic work and does not contain any original clinical or experimental data. All arguments and analyses presented are based on publicly available peer-reviewed literature, which are appropriately cited in the references. No original dataset was generated for this manuscript, and thus no data are available for sharing.
